# GaMD simulations as an alternative in the TFE-water mixture description

**DOI:** 10.1007/s00894-023-05749-4

**Published:** 2023-10-31

**Authors:** Itzel Pérez-Trejo, Laura Dominguez

**Affiliations:** Departamento de Fisicoquímica, Facultad de Química, Universidad Nacional Autónoma de México, Mexico City, 04510 Mexico

**Keywords:** 2, 2, 2-Trifluoroethanol, IDP, Gaussian acelerated molecular dynamic simulation, TIP4PD

## Abstract

**Abstract:**

*Context:* 2,2,2-Trifluoroethanol has been widely used to study the structure and dynamic properties of intrinsically disordered proteins. Experimentally, it is known that TFE-water mixtures stabilize secondary structures of IDPs, and therefore, it allows the studying of conformational ensembles of these proteins. In the last decades, molecular dynamic simulations have helped study the IDPs’ conformational ensemble. Unfortunately, conventional MD requires very long simulation times to describe the properties of IDPs. Therefore, a variety of accelerated sampling techniques have been developed and employed. The TFE-water mixture arrangement description through MD has faced substantial difficulties since emulating the TFE nanocrowding at certain TFE:H$_2$O ratios (around 15–40% of TFE). In this work, we determine the most suitable conditions that reproduce experimentally reported properties of TFE-water mixtures. We compared the employment of conventional MD and GaMD simulations and various water parameters. Our results show that the combination of parameters that better reproduce the experimental information is the combination of the TIP4PD water model and GaMD simulations. Therefore, these conditions help accurately describe the structural ensemble of IDPs in TFE-water mixtures. *Methods:* Conventional MD and GaMD simulations were performed under AMBER 18 software. The TFE and water molecules were described using GAFF2 and a variety of water models, such as TIP3P, TIP4P2005, TIP4PD, and TIP5P, respectively. The systems were simulated a 100 ns at 298 K.

**Graphical abstract:**

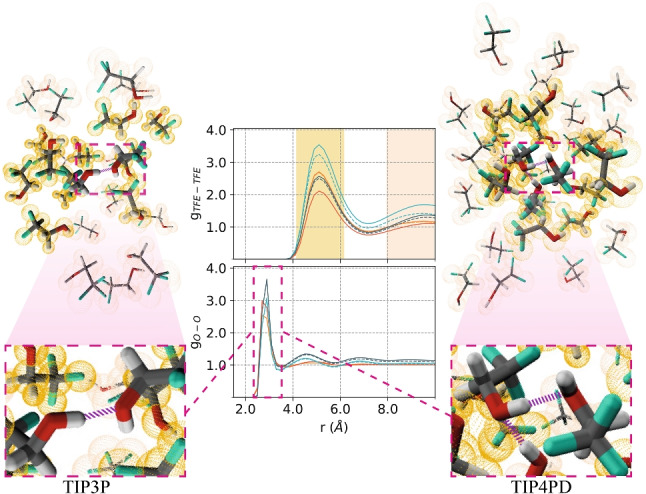

## Introduction

2,2,2-Trifluoroethanol (TFE) has been used as a cosolvent to characterize protein and peptide structures due to its ability to stabilize the secondary structure of proteins. Specifically, TFE molecules tend to preserve $\alpha$-helix [12] conformation of proteins; however, studies where TFE stabilizes $\beta$-harpins [2, 28] structures have also been reported. Additionally, it is well known that intrinsically disordered proteins (IDP) tend to get more ordered structures under TFE-water mixtures. TFE’s properties can contribute to the ordering of IDPs, such as the low dielectric constant, close to that of the interior of a protein; a low basicity [37], promoting the intramolecular hydrogen bonds of proteins; and its hydrophobicity [5] that may act as a protein denaturant.

To understand the ordering effect of IDPs under a TFE-water mixture, direct and indirect mechanisms of action have been proposed [18, 40]. In the direct mechanism, the TFE binds to helical peptide conformations modifying its natural interactions. Otherwise, the indirect mechanism suggests that TFE induces changes in the polypeptide solvent shell that accounts for the stabilization of helical structures [3]. Although different mechanisms of action suggest how TFE promotes ordering on IDPs, a proper mechanism must be described. Therefore, we carried out an atomistic description of the TFE-water mixture behavior by performing molecular dynamic (MD) simulations, where the TFE-water interactions have [26, 38] improved the TFE polarity by varying TFE parameters and water models [6, 17, 19, 39].

Molecular dynamic simulations are very helpful in understanding, at an atomic level, the ordering effect of IDPs under a TFE-water mixture. However, describing the proper nanocrowding of the TFE-water mixture remains challenging. Therefore, in this study, we compared different water parameters, force fields, and simulation methodologies commonly used to describe proteins and TFE-water mixtures. The description of water molecules is an important factor to consider when studying water-water and water-TFE interactions, as there are many water models, each focusing on different water properties. The TIP3P water model places the negative charge on the oxygen atom and the positive charge on the hydrogen atoms. TIP4P2005 is a model that was parameterized with the purpose of being a general model for the condensed phases of water. The TIP4PD water model aims to produce disordered state ensembles that are structurally compact by fitting the dispersion interactions. Finally, TIP5P is a model that accurately reproduces the density and radial distribution of liquid water.

Different types of simulations, such as temperature replica exchange molecular dynamics simulations (T-REMD) [29], have been performed to study conformational ensembles of IDPs. However, the use of these methodologies implies a high computational cost. To characterize the dynamic conformational ensemble of IDPs, the combination of both experimental and theoretical techniques [14, 25, 35, 36] have also been carried out.

In this study, we compared the use of MD and GaMD to determine if GaMD is a suitable methodology to study water-TFE mixtures. Gaussian accelerated molecular dynamics (GaMD) is a computational methodology used in molecular dynamics simulations that enhances the exploration of a complex conformational space. In GaMD, the potential energy surface is smoothed by adding a boost potential that follows a Gaussian distribution. The main advantage of using GaMD over MD simulations is that GaMD overcomes energy barriers and avoids only sampling local minima of the potential energy surface.

## Results and discussion

**The water model influences the aggregation properties of TFE molecules in a TFE-water mixture similarly in both MD and GaMD simulations.**

**Fig. 1:**
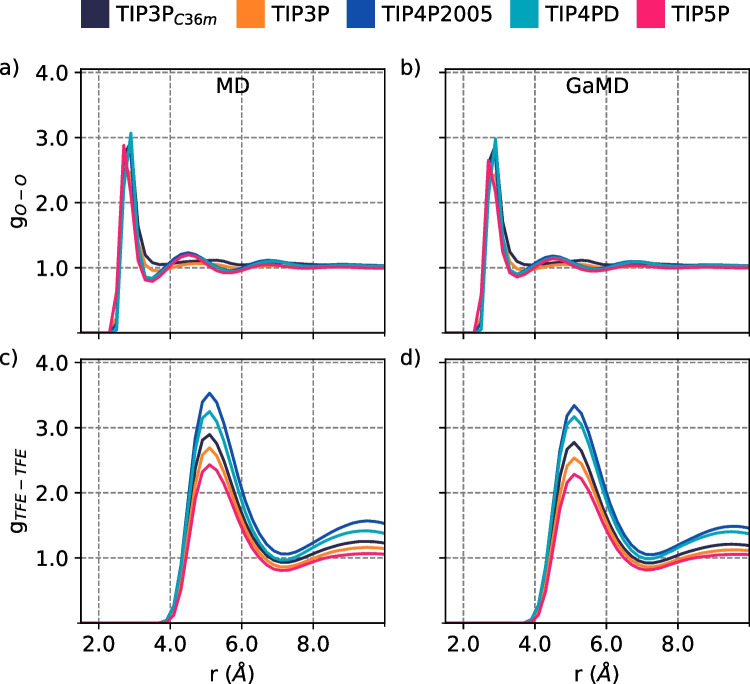
Oxygen-oxygen radial distribution function, between TFE−TFE molecules by **a** MD and **b** GaMD at 298 K, and center of mass $TFE-TFE$ radial distribution functions, for both methodologies **c** MD and **d** GaMD, by varying the water model

We employed MD and GaMD simulations in combination with different water models to evaluate the parameters that better describe the TFE-water mixture properties at different TFE:H$_2$O ratios. First, we intended to validate the hydrogen bond formation between TFE molecules; therefore, we calculated the trifluoroethanol oxygen-oxygen radial distribution function (g$_{O-O}$) in the TFE-water mixtures (Fig.  1 a and b). For all p1s simulated systems with the five different water models, we found a maximum value in the g$_{O-O}$ at a distance range between $r= 2.7$ and 2.9 *nm* at the $xTFE=0.1071$ mol fraction of TFE. This maximum value results from the hydrogen bond formation between oxygen atoms from the TFE-TFE interactions.

The p1s simulated systems with the TIP4P2005, TIP4PD, and TIP5P water models presented a broad peak in the g$_{O-O}$ between 4 and 6 *nm*, which corresponds to the distance between the neighboring TFE hydroxyl groups that are not forming a hydrogen bond. This broad peak indicates that the TFE molecules arrange themselves into an ordered structure, which is consistent with experimental data reported for pure TFE [33].

From the simulations, we found that the structural order and size of the TFE aggregates are influenced by the water model used, according to the center of mass TFE-TFE radial distribution function (g$_{TFE-TFE}$). The maximum peaks in the g$_{TFE-TFE}$ at around $r=5$ and $r=9.5$ *nm* (Fig. 1 c and d) indicate a favorable contact interaction between TFE-TFE molecules associated into clusters, in agreement with theoretical [7, 9, 11], and experimental [33] data, where the TIP5P water model provides a low aggregation propensity between TFE molecules. A more significant aggregation propensity was found with the TIP4P2005 water model; with the TIP4P2005, TIP4PD, and TIP5P water models, the oxygen atoms of TFE molecules are closer to other TFE molecules than with TIP3P, according to the results obtained by g$_{O-O}$ and g$_{TFE-TFE}$ (Table 1).

**Table 1:** Summary of simulation runs for the different system sizes and TFE:H$_2$O ratios

System	Force field	Water model	$x_TFE$	$N_TFE$	$N_wat$	% *v*/*vTFE*
p1s	C36M	TIP3P		306	2550	
		TIP3P				
p1b		TIP4PD	0.1071	1224	10,200	32.5
		TIP3P				
p2s		TIP4PD		845	2010	
		TIP3P				
p2b	GAFF2	TIP4PD	0.2960	3380	8040	62.8

In addition, the four-point water models, and particularly TIP4PD, exhibit densities that agreed well with the experimental data reported for the TFE-water mixtures [10, 31]. The calculated and experimental density and diffusion coefficient values for the different mixtures are reported in Table 2. In contrast, we found that the TFE diffusion coefficient values strongly depend on the water model and the type of simulation employed. Generally, the TFE diffusion coefficient values are higher in GaMD simulations than in conventional MD simulations. Additionally, we found that, by using either MD or GaMD dynamics, the diffusion values of TFE molecules are overestimated in the three-point water models. The closest TFE diffusion value to the reported experimentally [13] for the MD simulation is obtained by using the TIP5P water model. On the contrary, a similar value to experimental data using GaMD was obtained by performing a simulation with the TIP4PD water model.

**Fig. 2:**
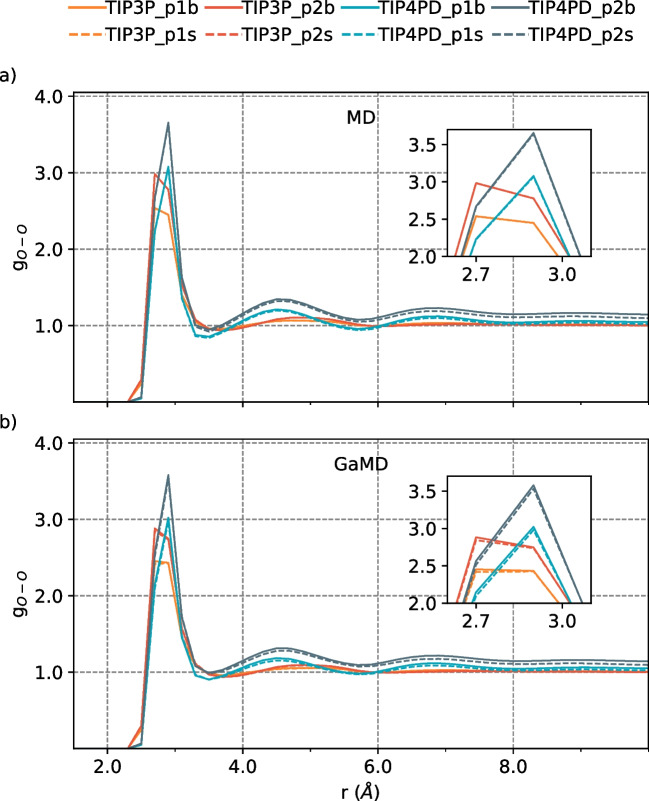
Oxygen-oxygen radial distribution functions between TFE-TFE molecules performed by **a** molecular dynamic (MD) and **b** Gaussian accelerated molecular dynamic (GaMD)

By contrasting the effect of the ratio on the description of TIP4PD and TIP3P water models, we found three maxima in the g$_{O-O}$ with TIP4PD, while for the TIP3P model, we only found two maxima (Fig. 2). This difference indicates that TIP4PD primarily exhibits neighboring TFE hydroxyl groups without forming hydrogen bonds; therefore, heterogeneity in the mixture is less observed with the four-point water model. Also, density values obtained by TIP4PD (Table 3) have greater coherency with experimental data [10, 31].

To understand if the system size and the TFE:H$_2$O ratio affect the TFE-water mixture dynamics, we performed additional simulations with a larger system size and a larger TFE:H$_2$O ratio, p1b, p2s, and p2b systems, by using the TIP3P and TIP4PD water models. We selected TIP3P since it is less computationally expensive and TIP4PD since it showed better agreement with experimental information.

**Table 2:** A comparison between density and diffusion coefficient for different water models for $xTFE=0.1071$ molar fraction

Model	Density g/cm$^3$		Diff. coeff. $10^{-9} m^2/s$	
	MD	GaMD	MD	GaMD
TIP3P	1.1087	1.0855	1.1618	1.3724
TIP4P2005	1.1345	1.1189	0.5233	0.8378
TIP4PD	1.1348	1.1203	0.4971	0.6644
TIP5P	1.1266	1.1029	0.6228	0.9047
C36m TIP3P	1.1296	1.1057	1.0755	1.344

**Fig. 3:**
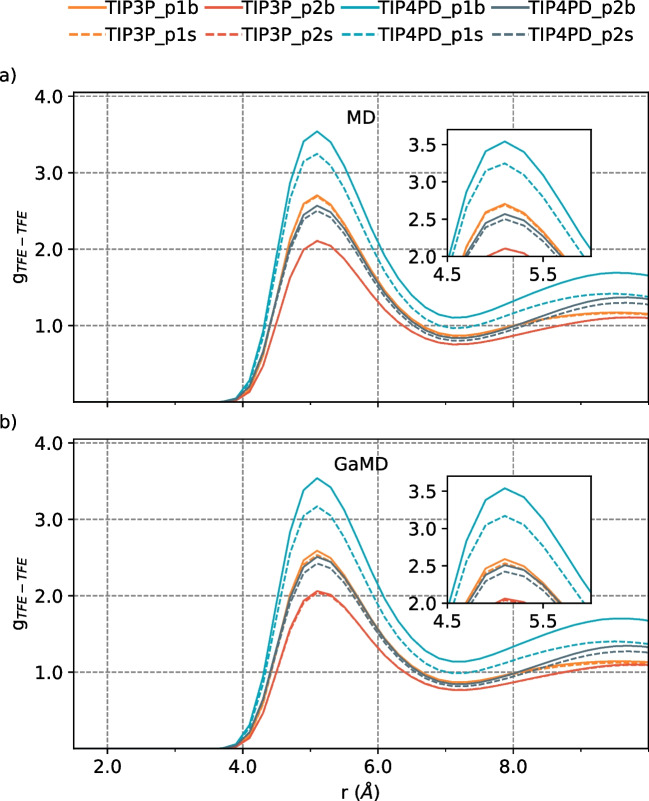
Center of mass $TFE-TFE$ radial distribution functions performed by **a** conventional MD and **b** GaMD simulations, by varying the TFE:H$_2$O ratio and system sizes

**Fig. 4:**
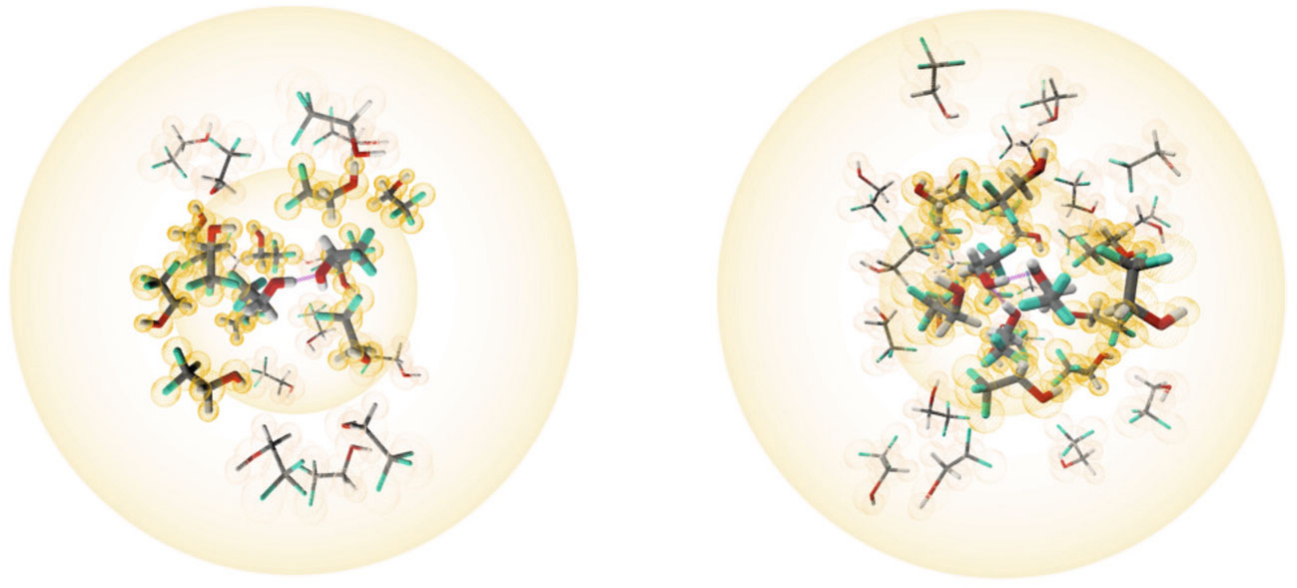
Spacial distribution of TFE molecules in TFE-water mixture with TIP3P (left) and TIP4PD (right) water models. Hydrogen bond formation and the first and second TFE solvation shells around it

**Oxygen-oxygen interactions between TFE molecules increase while increasing the TFE:H**$_{2}$**O ratio.** However, the number of TFE molecules in the aggregates does not increase but decreases at larger TFE:H$_2$O ratios. The latter suggests an increased heterogeneity of the mixture as the TFE:H$_2$O ratio increases. Due to a major availability of TFE molecules in the largest TFE:H$_2$O ratio, there is a slight increase in oxygen-oxygen interactions between TFE molecules. The TFE molecules with intramolecular H-bonding can form two intermolecular H-bonded contacts, where small cluster formation occurs with two or three molecules. However, the coordination number of TFE molecules decreases as the TFE:H$_2$O ratio rises. This finding has been previously reported in the literature [7]. And the observed behavior is more noticeable with TIP4PD compared to TIP3P. Also, TIP4PD presents two peaks in g$_{O-O}$ that indicate a less homogeneous system than TIP3P (Fig. 2) as the TFE:H$_2$O ratio increases. As a result, when the TIP4PD water model is employed, the number of aggregates of TFE molecules is larger than when employing TIP3P at larger ratios (Fig. 3). Moreover, the number of neighbor TFE molecules is higher in the first and second solvation shells (Fig. 4).

**Table 3:** Density values for each TFE:H$_2$O ratio and system size of the TFE-water mixture

System	TIP3P		TIP4PD	
	MD	GaMD	MD	GaMD
p1 s	1.1087	1.0855	1.1348	1.1203
p1b	1.1086	1.0923	1.1333	1.1239
p2 s	1.2222	1.1941	1.2525	1.2300
p2b	1.2221	1.2024	1.2513	1.2364

The diffusion coefficient values of TFE present a slight dependency on the TFE:H$_2$O ratio (Table 4). The diffusion coefficient values slightly increase as the molar ratio of TFE decreases; therefore, the TFE mobility is more significant at low concentrations of TFE molecules. Also, the diffusion coefficient results are in accord with density results. The TFE mobility is greater when TIP3P is employed due to the weaker mixture interaction by the water model’s poor polarity representation. Instead, in the simulations of the mixture with the TIP4PD water model and GaMD simulations, we obtained a better consistency between the diffusion coefficient values obtained in this work and the experimentally reported values. In the simulations employing the TIP4PD water model, we hypothesize that the enlarged water polarity causes the diminishing value of the diffusion coefficient.

**Table 4:** Diffusion coefficient values of TFE ($10^{-9}m^2/s$) of each TFE:H$_2$O ratio and system sizes, fraction mol xTFE = 0.29597

System	TIP3P			TIP4PD		
	MD		GaMD	MD		GaMD
p1 s	1.1618		1.3724	0.4971		0.6644
p1b	1.1388		1.3679	0.4479		0.6028
p2 s	0.9244		1.2537	0.4801		0.7293
p2b	0.9649		0.9649	0.4975		0.6533
		xTFE= 0.29167			0.610	
Exp. $^{1}$		xTFE= 0.32731			0.614	

$$^{1}$$Experimental data [13]

**Fig. 5:**
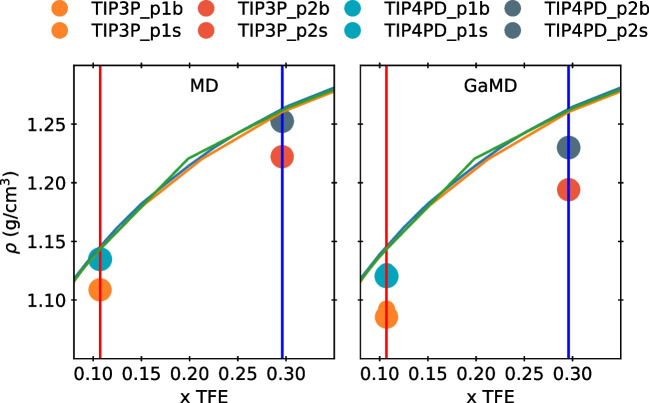
Density values for the TFE:H$_2$O ratios and system sizes. The TFE:H$_2$O ratios evaluated are shown in vertical lines, and solid lines are experimental data [10, 31]

**The system size did not affect TFE aggregation properties in the simulated systems.** As expected, the system size does not change the amount of oxygen-oxygen interactions between TFE molecules, as hydrogen bond interactions remain equal in both systems. However, we observed a slight difference in the center of mass TFE-TFE radial distribution function with the smallest TFE:H$_2$O ratio in combination with the TIP4PD water model (Fig. 3). However, there is no considerable system size effect on the aggregation of the TFE molecules or the system’s density.

**The employment of MD or GaMD did not affect the TFE-water mixture properties or dynamics. However, both methodologies slightly underestimate the density compared to the experimental values.**

We found that the tendency of the oxygen-oxygen radial distribution results between TFE molecules is not affected by the methodology used (Figs. 1, 2, and 3). Moreover, with both MD and GaMD dynamic types, at around $r=2.8$ $\text{\AA }$, hydrogen bond formation between TFE molecules is observed. Also, the center of mass TFE-TFE radial distribution functions show the same tendency for both MD and GaMD methodologies.

Both methodologies underestimate the experimentally reported density values (Fig. 5). The GaMD simulated systems combined with the TIP4PD water model also resulted in more homogeneous mixtures without losing the TFE aggregates. Although the diffusion coefficient obtained in the simulations better corresponds to the experimental data when the GaMD simulation is used, there are no significant differences in the rest of the properties of the mixture when either MD or GaMD methodologies are employed. Thus, we demonstrate that GaMD simulations could reproduce experimental information of a TFE-water mixture and could be further used to sample a protein’s structural ensemble in this hydrophobic environment with a lower computational cost than a conventional MD simulation.

## Methods

We performed MD and GaMD simulations to contrast the behavior of TFE-water mixtures through these two different types of simulations. Moreover, we combined the TIP3P [21], TIP4P2005 [1], TIP4PD [32], and TIP5P [27] water models with GAFF2 and C36m [15] parameters for selecting the more suitable description for the TFE-water mixtures. To compare the different water models, we used a small TFE:H$_2$O ratio (p1s system), which contains 306 and 2550 TFE and water molecules, respectively.

Once the water model was selected, we examined the effects of the system size and a different TFE:H$_2$O ratio with MD and GaMD simulations. For this, we built a system with a similar size to the original systems with a larger TFE:H$_2$O ratio (p2s system) that contained 845 TFE and 2010 water molecules; for these, we only used TIP3P and TIP4PD water models. The larger system size of each TFE:H$_2$O ratio (p1b and p2b systems) was built by adding four times the number of corresponding molecules. Table 1 summarizes the different system details. The initial configuration of all systems was obtained by adding the corresponding number of water and TFE molecules in a cubic cell through Packmol code [30]. The antechamber module [16] was used to build the parameter set for TFE, which is described by GAFF2. We employed four water models, TIP3P, TIP4P2005, TIP4PD, and TIP5P, to identify the most suitable water model to describe the TFE/water mixture; for this, we used the smaller system with the smaller TFE:H$_2$O ratio p1s.

In addition, the p1s$_{C36m-TIP3P}$ system was built with a CHARMM GUI code [20] in conjunction with the ligand reader and modeler input generator [22] for AMBER [24]. The C36m [15] parameters were employed for the TFE molecules in this system.

Classical molecular dynamics (MD) and Gaussian accelerated molecular dynamics (GaMD) simulations were carried out in the AMBER18 [4] simulation package. The systems were optimized by 1000 steps with the steepest descent and by its conjugate gradient until convergence was reached. Then, the volume of the system was adjusted under the NPT ensemble for 0.2 ns. The systems were then heated to 298 K in 50 ps and equilibrated for 3.95 ns. Subsequently, each system was submitted to a 100 ns production run using a 2 fs timestep. Hydrogen atoms were constrained using the SHAKE [34] algorithm.

The Ewald PME algorithm settled non-bonded interactions [8] with 8 $\text{\AA }$ cutoffs in real space. The temperature was regulated by employing Langevin dynamics [23] with a collision frequency of 2.0 ps$^{-1}$, and the pressure was controlled using the Berendsen barostat.

## Conclusion

In this work, we determined the best parameters to reproduce experimental information of TFE-water mixtures through conventional MD and GaMD. For this, we calculated the center of mass TFE-TFE and oxygen-oxygen radial distribution functions, system densities, and diffusion coefficients of the TFE-water mixtures. Initially, we varied the water model to select the one that better agreed with experimental and theoretical information. We also found that changes in the TFE:H$_2$O ratio and the system size did not affect the system TFE-water properties and dynamics. The employment of either MD or GaMD methodologies did not impact the simulations’ results presented in this work.

We found that by using the TIP4PD water model, we achieve more similar results to available experimental data, either by MD or GaMD simulations. Furthermore, considering that there is no significant effect on the TFE-water mixture properties when changing the type of simulation, being MD or GaMD, we demonstrate that it is possible to use GaMD simulation methodologies to study the hydrophobic effect of proteins or IDPs under the TFE-water mixture conditions.

## Data Availability

The datasets generated and/or analyzed during the current study are available from the corresponding author upon reasonable request.
